# Therapeutic efficacy of alpha-1 antitrypsin augmentation therapy on the loss of lung tissue: an integrated analysis of 2 randomised clinical trials using computed tomography densitometry

**DOI:** 10.1186/1465-9921-11-136

**Published:** 2010-10-05

**Authors:** Robert A Stockley, David G Parr, Eeva Piitulainen, Jan Stolk, Berend C Stoel, Asger Dirksen

**Affiliations:** 1 Lung Investigation Unit, University Hospitals of Birmingham, Edgbaston, Birmingham B15 2TH, UK; 2 Department of Respiratory Medicine, University Hospitals of Coventry and Warwickshire, Clifford Bridge Road, Coventry CV2 2DX, UK; 3 Department of Respiratory Medicine, Malmö University Hospital, Lund University, Malmö, 205 02, Sweden; 4 Leiden University Medical Center, Albinusdreef 2, 2333 ZA Leiden, The Netherlands; 5 Gentofte Hospital, Copenhagen University, DK-2900 Hellerup, Denmark

## Abstract

**Background:**

Two randomised, double-blind, placebo-controlled trials have investigated the efficacy of IV alpha-1 antitrypsin (AAT) augmentation therapy on emphysema progression using CT densitometry.

**Methods:**

Data from these similar trials, a 2-center Danish-Dutch study (n = 54) and the 3-center EXAcerbations and CT scan as Lung Endpoints (EXACTLE) study (n = 65), were pooled to increase the statistical power. The change in 15^th ^percentile of lung density (PD15) measured by CT scan was obtained from both trials. All subjects had 1 CT scan at baseline and at least 1 CT scan after treatment. Densitometric data from 119 patients (AAT [Alfalastin^® ^or Prolastin^®^], n = 60; placebo, n = 59) were analysed by a statistical/endpoint analysis method. To adjust for lung volume, volume correction was made by including the change in log-transformed total lung volume as a covariate in the statistical model.

**Results:**

Mean follow-up was approximately 2.5 years. The mean change in lung density from baseline to last CT scan was -4.082 g/L for AAT and -6.379 g/L for placebo with a treatment difference of 2.297 (95% CI, 0.669 to 3.926; p = 0.006). The corresponding annual declines were -1.73 and -2.74 g/L/yr, respectively.

**Conclusions:**

The overall results of the combined analysis of 2 separate trials of comparable design, and the only 2 controlled clinical trials completed to date, has confirmed that IV AAT augmentation therapy significantly reduces the decline in lung density and may therefore reduce the future risk of mortality in patients with AAT deficiency-related emphysema.

**Trial registration:**

The EXACTLE study was registered in ClinicalTrials.gov as 'Antitrypsin (AAT) to Treat Emphysema in AAT-Deficient Patients'; ClinicalTrials.gov Identifier: NCT00263887.

## Introduction

In subjects with a hereditary deficiency of alpha-1 antitrypsin (AAT), the pathophysiology of emphysema is believed to be a direct consequence of tissue damage caused by a reduced ability of AAT to inactivate neutrophil elastase, which is released by migrating neutrophils in response to inflammatory stimuli [1]. It is logical that augmentation of the circulating levels (and hence lung levels) of AAT would confer normal protection by restoring the inhibitory capacity of AAT in the lungs. The net result is argued to be retardation of the destructive process and, therefore, the progressive decline in lung physiology [2]. A strategy of weekly augmentation with AAT was thus introduced in the 1980s, confirming that the attainment of a putative protective level was possible with weekly infusions of AAT at a dose of 60 mg•kg^-1 ^body weight [3].

Because the numbers required to perform a controlled clinical trial using forced expiratory volume in 1 second (FEV_1_) are thought to be prohibitive (requiring inclusion of a large number of individuals with a rare disease over many years [4,5]), no such study has been undertaken. Despite this, augmentation therapy is widely prescribed using varying treatment intervals and doses of plasma-derived AAT [6].

In the past, the mainstay of clinical assessment of emphysema was lung function and especially gas transfer measurements, although recent data have indicated that there is differential progression depending on disease severity [7]. Computed tomography (CT) densitometry is a validated and more direct measure of pathological emphysema [8-10] that relates well to physiological and clinical features of disease [11,12], progresses uniformly across disease severity [10] and has specifically been shown to be the best independent predictor of mortality [13].

In 1999, Dirksen, *et al *reported a 3-year Danish-Dutch controlled study of intravenous (IV) AAT augmentation therapy, with loss of lung tissue measured by CT densitometry as a secondary outcome parameter in 56 patients [14]. The study suggested a reduction in emphysema progression with AAT augmentation therapy measured by CT, although the p value for the treatment difference obtained (p = 0.07) failed to achieve the conventional level of significance, which may reflect the number of subjects in the trial.

More recently, the EXAcerbations and CT scan as Lung Endpoints (EXACTLE) study (77 patients studied over 24-30 months), using a similar placebo-controlled trial design of IV AAT, explored CT densitometry as the primary outcome [15]. Lung density was analysed using 4 different methods of adjustment that corrected for variation in inspiratory levels between scans, and all showed a trend towards efficacy. However, endpoint analysis using a statistical correction for lung volume not only proved to be the most sensitive method of analysis (based on monitoring progression in the placebo group), but also achieved a conventional level of statistical significance with regard to lung tissue loss between both treatment groups. Interestingly, in both the Danish-Dutch and EXACTLE studies, there was little difference in density loss between the AAT and placebo groups within the first year while, subsequently, the difference between the groups increased with time. Furthermore, the effect of therapy in clinical trials is usually determined by endpoint analysis. For these reasons, we chose to re-analyse the Danish-Dutch study using an endpoint analysis, utilising only the first and last available measurement.

In addition, because of the similar study design and method of CT densitometry, we combined the raw data from both studies to increase the statistical power as suggested in the previous Danish-Dutch study [14].

## Materials and methods

Characteristics of the study subjects and designs of the Danish-Dutch and EXACTLE studies are presented in Table 1. Full methodological details, together with further details of the patient inclusion and exclusion criteria for the 2 studies, can be found in the original publications [14,15].

**Table 1:** Comparison of study characteristics

	Danish-Dutch trial	EXACTLE trial
Genotype/phenotype	PiZZ on IEF	PiZZ or severe deficiency with AAT concentrations <11 μM
Lung function, FEV_1_	30-80%	25-80% and FEV_1_/VC ≤70% or
K_co_	NA	≤80% if spirometry normal
Exacerbations	NA	≥1 exacerbation in the past 2 years
Smoking history	Never or ex-smokers for >6 months Cotinine checked every 4 weeks	Never or ex-smokers for >6 months Cotinine checked at 1, 6, 24 and 30 months
Previous augmentation therapy	NA	Never or ≤1 month in past 2 years
Study design	Randomised, double-blind, placebo-controlled	Randomised, double-blind, placebo-controlled
AAT dosing	250 mg•kg^-1 ^body weight AAT	60 mg•kg^-1 ^body weight AAT
Treatment interval	Every 4 weeks	Every week
Placebo	625 mg•kg^-1 ^body weight albumin	2% albumin
Centres	2 (Copenhagen, Leiden)	3 (Copenhagen, Birmingham, Malmö)
Duration of study	Minimum 3 years	24 months (optional 6-months extension)
Study period	January 1991 to August 1997	November 2003 to December 2006
Primary endpoints	FEV_1 _measured by home spirometry twice daily	Change in PD15 measured by CT
Other endpoints	Change in PD15 measured by CT	Exacerbations Lung function (FEV_1_, K_CO_) Quality of life (SGRQ)

AAT: alpha-1 antitrypsin; EXACTLE: Exacerbations and Computed Tomography scan as Lung Endpoints; IEF: isoelectric focusing; K_co_: carbon monoxide transfer coefficient; NA: not applicable; PD15: 15^th ^percentile lung density; SGRQ: St George's Respiratory Questionnaire; VC: vital capacity. CT: computed tomography.

### Patients

**Figure 1:**
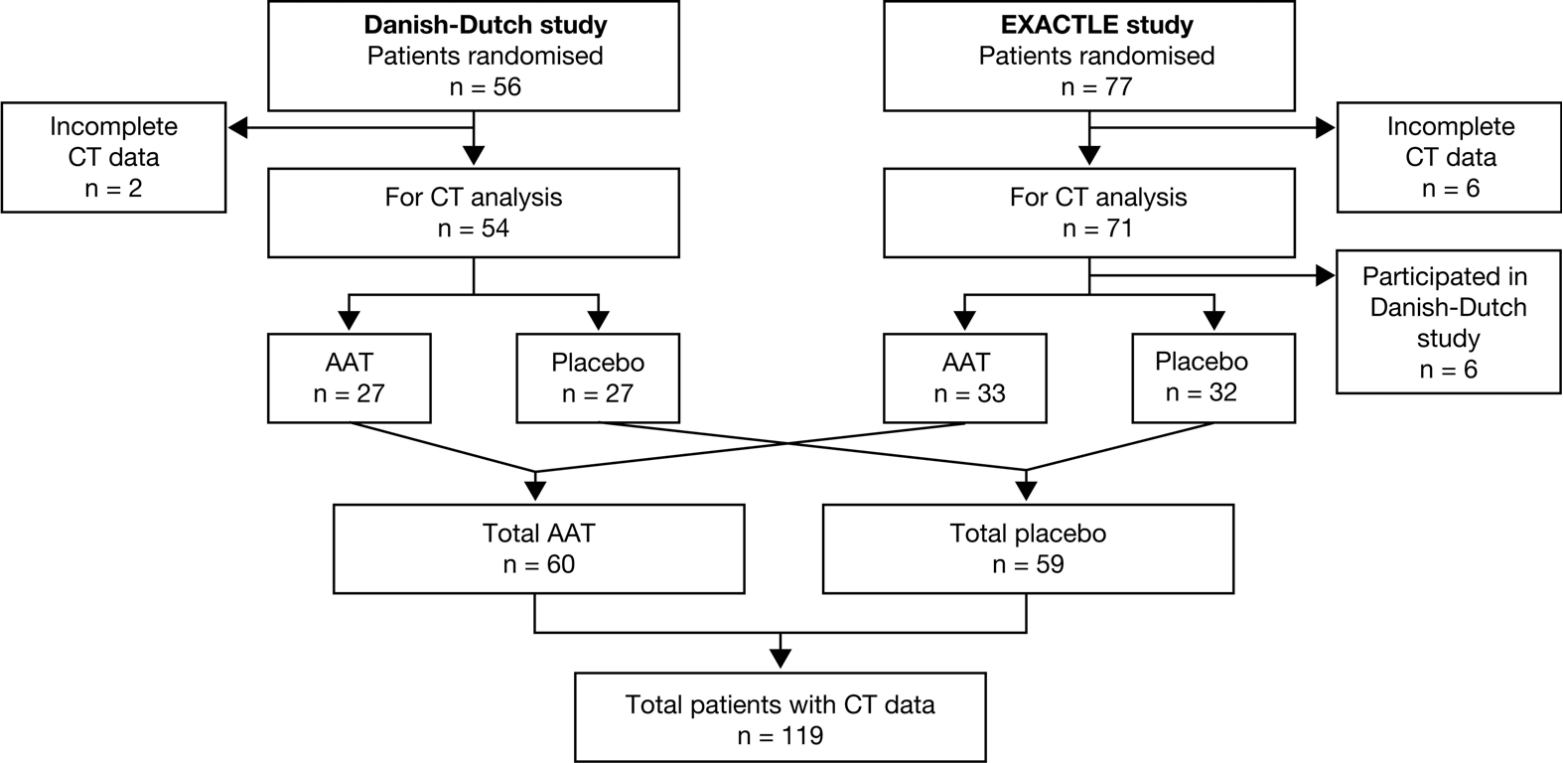
**Patient disposition by treatment (patients providing CT data)**. AAT: alpha-1 antitrypsin; EXACTLE: Exacerbations and Computed Tomography scan as Lung Endpoints.

Pooled patient data from the 2 previously described trials, the 2-centre Danish-Dutch study (Copenhagen, Denmark; Leiden, The Netherlands) [14] and the 3-centre EXACTLE study (Copenhagen, Denmark; Birmingham, United Kingdom; Malmö, Sweden) [15], are summarised in Table 2. All patients had been recruited from AAT deficiency registries. The Danish-Dutch study randomised 56 patients and there were 77 from EXACTLE; in total, 125 patients were valid for CT data analysis (Figure 1). However, 6 patients originally enrolled in the Danish-Dutch trial also participated in the EXACTLE study. The data for these 6 subjects from EXACTLE were therefore excluded from the integrated analysis. The original studies had been approved by local ethics committees and were conducted in accordance with the Declaration of Helsinki and Good Clinical Practice Guidelines.

**Table 2:** Patient baseline demographic characteristics*

	Danish-Dutch trial	EXACTLE trial	Combined data				
	**AAT** **(n = 27)**	**Placebo** **(n = 27)**	**AAT** **(n = 38)**	**Placebo** **(n = 39)**	**AAT** **(n = 60)**	**Placebo** **(n = 59)**	**p value**
Age (y)	48.0 ± 7.99	47.5 ± 7.29	54.7 ± 8.4	55.3 ± 9.8	51.6 ± 9.03	51.8 ± 9.73	0.808
Sex (n) male/female	18/9	16/11	25/13	16/23	38/22	29/30	0.093
Smoking status (n, ex/never)	27/0	27/0	34/4	35/4	56/4	56/3	0.748
Body mass index (kg•m^2^)	23.3 ± 3.15	24.4 ± 2.70	24.3 ± 3.3	24.3 ± 3.5	24.0 ± 3.3	24.5 ± 3.2	0.355
FEV_1 _(L), median	1.63 ± 0.49 1.63	1.72 ± 0.53 1.61	1.44 ± 0.60 1.14	1.35 ± 0.62 1.14	1.55 ± 0.56 1.47	1.48 ± 0.63 1.38	0.553
FEV_1_% predicted, median	47.3 ± 11.4 48.6	51.2 ± 14.5 49.0	46.3 ± 19.6 41.1	46.6 ± 21.0 39.5	48.0 ± 16.4 47.2	47.9 ± 18.6 43.1	0.949
	Danish-Dutch trial	EXACTLE trial	Combined data				
	AAT (n = 27)	Placebo (n = 27)	AAT (n = 38)	Placebo (n = 39)	AAT (n = 60)	Placebo (n = 59)	p value
VC % predicted	114 ± 14.7	117 ± 16.4	94 ± 21.8	98 ± 23.2	103.1 ± 21.8	104.7 ± 23.9	0.789
DLCO% predicted Median	59.7 ± 16.0 57.0	60.1 ± 16.3 65.0	50.7 ± 19.5 47.6	52.2 ± 15.2 50.1	56.3 ± 17.3 56.1	55.7 ± 15.9 56.0	0.797
KCO % predicted	62.2 ± 17.62	59.9 ± 16.9	55.3 ± 21.0	56.5 ± 14.8	60.0 ± 18.9	58.6 ± 15.5	0.619
Unadjusted PD15 (g•L^**-1**^)	71.41 ± 20.87	75.56 ± 25.53	47.98 ± 19.07	45.48 ± 16.95	58.88 ± 23.03	59.79 ± 25.83	0.844
TLC-adjusted PD15^† ^(g•L^**-1**^)	59.9 ± 11.03	62.98 ± 13.49	54.6 ± 17.4	53.9 ± 16.0	57.1 ± 15.2	58.2 ± 15.7	0.691
Lung volume (L)	5.71 ± 1.27	5.52 ± 1.34	7.46 ± 1.60	7.27 ± 1.78	6.61 ± 1.67	6.35 ± 1.69	0.300

* Values are mean ± SD unless otherwise indicated.

^†^TLC-adjusted PD15: CT lung density multiplied by CT-measured total lung volume and divided by the individual patient's predicted TLC.

For the CT densitometric analyses, the modified ITT population was used.

The combined analysis was based on the modified ITT population and did not include the data for 6 subjects who participated in EXACTLE, but who had also participated in the earlier Danish-Dutch study.

AAT: alpha-1 antitrypsin; DLco: diffusion capacity of the lung for carbon monoxide; EXACTLE: Exacerbations and Computed Tomography scan as Lung Endpoints; Kco: carbon monoxide transfer co-efficient; PD15: 15^th ^percentile lung density; TLC: total lung capacity; VC: vital capacity.

### Study designs

Both studies were randomised, placebo-controlled, double-blind, parallel-group trials [14,15]. Patients in the Danish-Dutch study were randomised to receive infusions of either AAT (Alfalastin^®^; Laboratoire Français du Fractionnement et des Biotechnologies, 3 avenue des Tropiques, BP 305, Les Ulis, 91958 Courtaboeuf Cedex, France; 250 mg•kg^-1 ^body weight) or placebo (human albumin solution; 625 mg•kg^-1 ^body weight) every 4 weeks for ≥3 years [14]. Patients in the EXACTLE study were randomised to weekly infusions of AAT (Prolastin^®^; Talecris Biotherapeutics, Inc., Research Triangle Park, NC, USA; 60 mg•kg^-1 ^body weight) or placebo (2% albumin) for 24 months, with an optional extension to 30 months in subjects who agreed to continue in the study [15].

### Data analysis and CT densitometry

**Figure 2:**
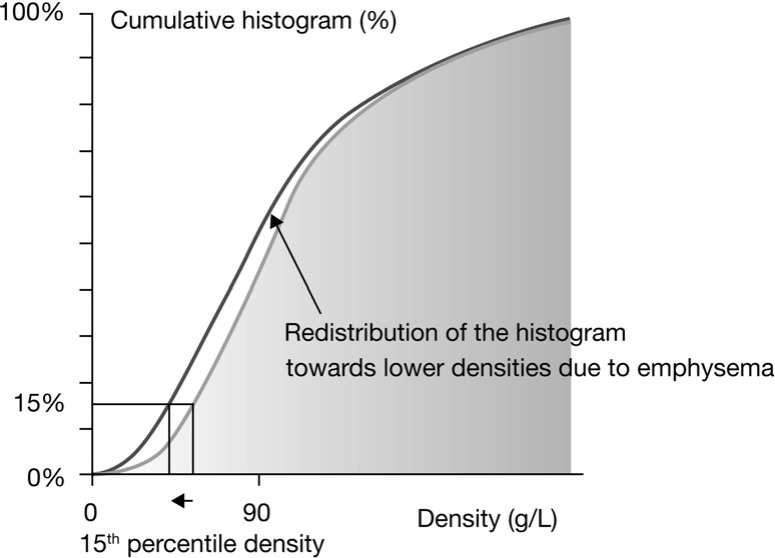
**Measurement of progression of emphysema**.

The rate of emphysema progression was determined by change in lung density measured by whole lung CT scan, and reported as the annual change in the 15^th ^percentile lung density (PD15) (determined from the endpoint in the original trials). The PD15 value is extracted from the frequency histogram of lung voxels and is the density value (g•L^-1^) at which 15% of the voxels have lower densities [9,10] (Figure 2). This analysis combines the raw data from both trials, thereby increasing the numbers of patients and the robustness of the analysis.

CT scans were performed at baseline and annually thereafter. In the EXACTLE study, there was an option for additional scans at 30 months in those subjects who had their participation prolonged from 24 months [15]. CT scans were obtained during both trials using different scanner protocols. For the Danish-Dutch study, scans were acquired during a breath hold (Dutch patients) or during quiet tidal breathing (Danish patients). The EXACTLE trial acquired scans during a breath hold at maximum inspiration as summarised in the online supplement for Dirksen *et al *[15]. In both trials, CT scanners were carefully calibrated and all scan data were centrally analysed by BioImaging Technologies, Inc. (Leiden, The Netherlands) using PulmoCMS^® ^(Medis Specials, Leiden, The Netherlands) for the EXACTLE study, and by Leiden University Medical Centre for the Danish-Dutch study.

### Data analysis and FEV_1_

We also took the opportunity to review the FEV_1 _decline from both studies using all available data and a slope analysis for the patients included in the integrated analysis. From the original Danish-Dutch study we were, however, unable to retrieve spirometry from 4 of the subjects.

### Volume correction of CT Scans

The level of inspiration during scan acquisition is recognised to influence lung density and reduce the reproducibility of CT. In the chosen method (statistical/endpoint analysis method), volume correction was made by including the change in log-transformed total lung volume (TLV) as a covariate in the statistical model as described [14]. This method corrects for intra-patient differences in inspiration between scans as well as inter-patient differences in technique between centres.

### Statistical analysis

The raw data from the Danish-Dutch and EXACTLE studies were retrieved and combined. A study ID variable was included in the integrated analysis database to identify the records in the Danish-Dutch or EXACTLE studies.

All CT scan analyses were based on the modified intent-to-treat (ITT) population, which included (in common with the ITT) all randomised subjects who received the study therapy. However, those subjects in the modified ITT population also had to have one valid CT scan measurement at baseline and at least one valid CT scan assessment at the Month 12 visit or after.

For the Danish-Dutch and EXACTLE studies, PD15 was analysed using an analysis of covariance (ANCOVA) model with change from baseline to the last CT scan measurement in PD15 as the dependent variable, treatment and centre as fixed factors, and change in logarithm of CT-measured TLV and baseline measurement as covariates (statistical/endpoint analysis method).

For the combined data of the integrated analysis, the study ID was added to the model as a fixed effect. The ANCOVA model included the change from baseline to the last CT scan as the dependent variable; study (EXACTLE versus Danish-Dutch), treatment, centre and change in logarithm of lung volume as fixed factors, and baseline measurement as covariate.

## Results

### Patient disposition and baseline characteristics

CT densitometric measurements from a total of 119 patients were analysed (AAT, n = 60; placebo, n = 59). In the Danish-Dutch study, CT data were obtained from 54 patients, comprising 26 patients from Denmark and 28 patients from The Netherlands. In the EXACTLE study, 65 patients provided data, 27 from Denmark, 23 from the United Kingdom and 15 from Sweden. The patient disposition by treatment is shown in Figure 1.

In the Danish-Dutch study, the mean (range) length of exposure was 2.52 (0.9-4.2) years to AAT, and 2.55 (0.9-3.9) years to placebo. The corresponding values in the EXACTLE study were 2.23 (1.1-2.6) and 2.18 (0.8-2.6) years, respectively. For the combined data from both studies, the mean (range) length of exposure to AAT was 2.36 (0.9-4.2) years and to placebo, 2.33 (0.9-3.9) years.

The characteristics for patients at baseline are summarised in Table 2. Baseline demographics for patients enrolled into the Danish-Dutch and EXACTLE studies were comparable, although patients in the EXACTLE study were slightly older and had a lower FEV_1_% predicted. For the combined data, there were no statistically significant differences between the group receiving AAT or placebo with respect to age or body mass index. There were some gender differences between the treatment groups, with more male subjects in the active treatment group, although this was not statistically significant (p = 0.093).

All patients fulfilled the physiological inclusion criteria shown in Table 1. There were no statistically significant differences at baseline between the treatment groups with regard to these parameters. There was also no significant difference in total lung capacity-adjusted PD15 between the 2 groups at baseline (p = 0.691).

### CT densitometric progression

**Figure 3:**
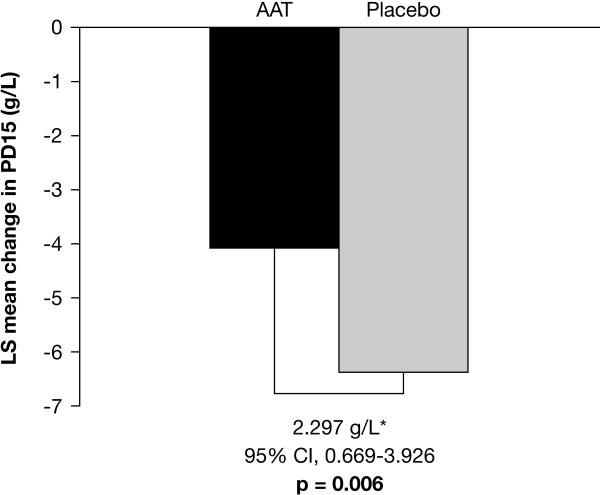
**Progression of emphysema in AAT-treated versus placebo-treated subjects (modified ITT)**. *Estimated treatment difference between mean changes in unadjusted 15^th ^percentile lung density from baseline. AAT: alpha-1 antitrypsin; LS: least squares; PD15: 15^th ^percentile lung density.

From the Danish-Dutch study, the least squares mean change in PD15 from baseline to endpoint was greater in the placebo group than in the active group (3.155; p = 0.049; Table 3). Combined data from the Danish-Dutch and EXACTLE studies confirmed the reduction in progression in patients receiving augmentation therapy (-6.379 g•L^-1 ^[placebo] versus -4.082 g•L^-1 ^[AAT]; p = 0.006; Figure 3), which is approximately equivalent to -2.74 and -1.73 g•L^-1^•yr^-1^, respectively. Therefore, using the most sensitive statistical/endpoint analysis method of volume correction, the separate and integrated analysis of the 2 trials demonstrated a significant reduction in the loss of lung tissue for subjects receiving treatment with IV AAT in comparison with those receiving placebo.

**Table 3:** Changes in unadjusted 15^th ^percentile lung density (g•L^-^^1^) using endpoint analysis

	Danish-Dutch trial	EXACTLE trial	Combined data			
**Statistic**	**AAT** **(n = 27)**	**Placebo** **(n = 27)**	**AAT** **(n = 36)**	**Placebo** **(n = 35)**	**AAT** **(n = 60)**	**Placebo** **(n = 59)**
Change from baseline to last CT scan, LS mean	-6.409	-9.564	-2.645	-4.117	-4.082	-6.379
Estimated treatment difference between changes from baseline, 95% CI^†^	3.155 (0.008-6.301)	1.472 (0.009-2.935)	2.297 (0.669-3.926)			
p value for treatment difference	0.049	0.049	0.006			

For the CT densitometric analyses, the modified ITT population was used. The combined analysis was based on the modified ITT population and did not include the data for 6 subjects who participated in EXACTLE, but who had their data included in the earlier Danish-Dutch study.

^†^AAT treatment minus placebo.

AAT: alpha-1 antitrypsin; CT: computed tomography; EXACTLE: Exacerbations and Computed Tomography scan as Lung Endpoints; LS: least squares.

### FEV_1 _decline

The FEV_1 _declined significantly in both the combined treated and placebo groups. The average annualised difference in FEV_1 _loss was 13 mL•yr^**-1 **^greater in the treated group although this is within the error of measurement (95% CI, -38 to 13; p = 0.321).

## Discussion

Until now, a suitably powered double-blind randomised trial of the clinical effectiveness of AAT augmentation therapy has been lacking. The individual and combined analysis of the Danish-Dutch and EXACTLE trials confirms that AAT augmentation therapy has a beneficial effect on the decline in lung density, which is a measure of the progression of emphysema.

AAT augmentation therapy is an accepted therapeutic regimen [6], and an earlier observational study showed better overall survival and reduced FEV_1 _decline (albeit in a subset with moderate airflow obstruction) for patients receiving therapy with varying regimens [16]. Whereas the recommended regimen is 60 mg•kg^-1 ^body weight per week, other adopted approaches are likely to be as effective if the nadir AAT level is mostly above the putative protective threshold of 11 μM.

Preservation of normal lung structure has been the long-term aim of preventive therapy in chronic obstructive pulmonary disease (COPD). However, studies of this concept have used FEV_1 _as the endpoint, since it is not only a defining feature of COPD but also reflects patients with a variety of phenotypes, including those with small airways disease and emphysema. Moreover, FEV_1 _is a reasonable marker of a patient's health status and exercise capacity [17], and has previously been considered to be the best predictor of respiratory and all-cause mortality [18]. This has led to the tenet that the maintenance of FEV_1 _reflects disease stability or a consequent reduction in mortality. Nevertheless, FEV_1 _is a poor surrogate measure for the presence and severity of emphysema and its progression. For instance, it has been demonstrated that patients with apical emphysema may have a preserved FEV_1 _in both AAT deficiency [8,19] and usual COPD [20].

The FEV_1 _data from this combined study confirm that even doubling the number of subjects is inadequate to verify whether augmentation therapy affects this non-specific and relatively insensitive outcome of emphysema. Much larger numbers of subjects studied over a longer period of time are required [4] in order to determine the response of therapy on FEV_1_, even though longitudinal CT data have confirmed that decline in FEV_1 _does generally relate to loss of lung density, but only if sufficient data are analysed [10]. Extensive observational studies of lung density in AAT deficiency using CT scanning have demonstrated that this parameter not only relates to progressive reduction in FEV_1 _[10], health status and exercise capacity [11], but is indeed a better predictor of all-cause mortality than FEV_1 _[13]. It is possible to extrapolate the findings of this combined analysis to conventional measures such as the FEV_1 _using previously published data [10]. This indicates that the reduction in densitometry quantified here (ྜ1 HU/year) is equivalent to about a 38 ml difference in FEV_1 _decline in patients in GOLD stage 2.

However as indicated above the decline in FEV_1 _is not linear throughout the disease process. Therefore, for this and other reasons, stabilisation of emphysema progression, as indicated by CT densitometry, would be as important an aim, if not more so, than preserving FEV_1_. The current combined analysis of the only 2 controlled clinical trials completed to date has confirmed that AAT augmentation therapy significantly reduces the decline in lung density, and may thus reduce the future risk of mortality as well as the deterioration in health status.

With AAT augmentation therapy becoming widely accepted throughout the United States and Europe, the ability to deliver appropriately powered placebo-controlled clinical trials, particularly those requiring a physiological measurement outcome, has become difficult to justify ethically and even more difficult to deliver. The current analysis, however, provides evidence of augmentation therapy reducing the rate of progression of lung tissue loss. The data, therefore, permit future studies to be powered for comparison of different therapeutic regimens using CT scans rather than physiology (either FEV_1 _or gas transfer). However, it should also be noted that even CT scans, as well as accepted physiological measurements, are only surrogate measures of emphysema. Importantly, the change in physiological endpoints varies throughout the course of the disease, with FEV_1 _decline being greatest in subjects with moderate airflow obstruction (35-79% of predicted) [16] and gas transfer decline being greatest in those with most severe disease [7]. On the other hand, loss of lung density as assessed by PD15 shows a more constant change across all stages of disease severity [10], suggesting that it is a better marker of the continuing disease process.

It is not always feasible to conduct powered clinical studies [21], and sometimes a combination of comparable studies is necessary. For example, meta-analysis of several studies has been used to support the use of antibiotics in acute exacerbations of COPD [22].

In clinical medicine, meta-analyses are accepted and useful tools that combine results from several studies to draw conclusions about clinical effectiveness. These can be either based on the analysis of published data (so-called 'aggregated analysis') or by pooling individual patient data (also termed 'integrated analysis') [23]. Trials with different protocols, but with common characteristics, can be pooled for these analyses. An integrated analysis based on individual patient data offers numerous advantages over the use of aggregated data; it is more reliable than aggregate meta-analyses and may thus lead to different conclusions [23,24]. This approach has been used more frequently in recent years [24] and also allows, as aggregate analyses similarly do, for the inclusion of different drug substances belonging to the same drug class, and different predefined clinical endpoints in the source studies [25,26], provided that the studies have common characteristics to enable the pooling of data.

Although there were some differences in study characteristics, the EXACTLE and Danish-Dutch trials both had a randomised, placebo-controlled, blinded, parallel design and had a similar CT scan protocol. The 2 studies were comparable with regard to treatment drug, treatment duration and patient characteristics. There is a general belief that maintaining AAT above a protective level of 11 μM is the key to a successful therapeutic outcome, and both studies had treatment regimens that are able to maintain protective levels of AAT, either consistently, or for at least 3 out of the 4 weeks in the monthly regimen used in the Danish-Dutch trial [14].

The Jadad scale is widely used to assess the methodological quality of clinical trials [27,28]. When evaluated on this scale, the design of the 2 studies met the standards required for their results to be included in a meta- or integrated analysis. Although the principle of meta- or integrated analyses is based on the inclusion of several studies, p values are reported without statistical adjustment of the alpha level.

Integrating the data from the 2 studies increased the numbers and hence the power of the observations. By using the most sensitive method for assessing emphysema progression (as measured by tissue loss) with endpoint analysis of PD15, the mean data demonstrate a deceleration of lung tissue loss with AAT augmentation therapy with a high degree of statistical significance. It is, however, recognised that progression even in CT densitometry varies between individuals. Thus adequate historical data will remain a prerequisite to therapeutic decision making. Furthermore, it should be noted that the treatment effect may not be demonstrable for the first 12 months of therapy [14,15]. The exact reasons remain unknown but it is possible that a period of time is required to reverse the established, destructive inflammatory process. This observation clearly has potential impact on the design of future phase 2 and 3 studies in AAT deficiency and support an end point analysis as the best primary outcome.

In conclusion, the overall results are supportive of the efficacy of AAT augmentation therapy and, importantly, provide confirmatory data to power and analyse future alternative strategies for which long-term IV placebo arms cannot be justified ethically.

### Disclosure of prior abstract publications

Abstracts of this study have been published by the American Thoracic Society (*Am J Respir Crit Care Med*, Apr 2008;177), and by the European Respiratory Society (*Eur Respir J*, Oct 2008;32(Supplement 52):738s).

## Competing interests

Robert A Stockley has received an unrestricted grant from Talecris Biotherapeutics for the Alpha-1 Detection and Programme for Treatment (ADAPT UK registry). He has advised Baxter and Kamada on their augmentation programmes and received international lecture fees from Talecris. He has lectured widely as part of pharmaceutical sponsored symposia, sat on numerous advisory boards for drug design and trial implementation and received non-commercial grant funding from some companies. David G Parr has served on company advisory board meetings for Talecris Biotherapeutics and acts as a consultant on the technical steering committees of Talecris Biotherapeutics and F Hoffmann-La Roche. He has received honoraria and payment of expenses from Talecris Biotherapeutics for presentations at international meetings. Eeva Piitulainen has no conflicts of interest to disclose. Jan Stolk has served on company advisory board meetings of various companies and served as consultant to some of them. Fees were directly donated to the bank account of the Alpha-1 International Registry Foundation. Berend C Stoel has received honoraria for presentations from Talecris Biotherapeutics. He is a consultant for Roche Pharmaceuticals, Talecris Biotherapeutics, Bioclinica and CSL Behring. His institution has received grant monies from Bio-Imaging (now Bioclinica), Roche, Talecris and Medis Medical Imaging Systems for a research project. Asger Dirksen, as the principal investigator of the 2 multicenter, randomised clinical trials of augmentation therapy with alpha-1 antitrypsin that are integrated in the manuscript, has received grant monies from Bayer and Talecris Biotherapeutics, and has participated in travel and meetings sponsored by Bayer and Talecris. Furthermore, he has received grant funding from the Danish Lung Association for a PhD, who shall analyse data from the Danish Lung Cancer Screening Trial that has no relation to the manuscript.

## Authors' contributions

RAS was an investigator in the EXACTLE study and proposed the combined analysis. He wrote the first draft of the manuscript and has fine-tuned the final version, following input from all co-authors and with subsequent support from a medical writer. DGP has been involved in the methodology for CT analysis of the EXACTLE study and the integrated data. He has revised the submitted article for important intellectual content, and has approved the final version. EP was responsible for the Swedish arm of the EXACTLE study. She has reviewed and approved the manuscript. JS was an investigator in the Dutch part of the Danish-Dutch study and was involved in the design of the EXACTLE study. He has revised the submitted article critically for important intellectual content, and has provided final approval of the version to be published. BCS has been involved in the methodology for CT analysis used in both studies. He has revised the submitted article critically for important intellectual content, and has provided final approval of the version to be published. AD was the principal investigator of the 2 multicentre, randomised clinical trials of augmentation therapy with AAT. He has revised the submitted article critically for important intellectual content, and has provided final approval of the version to be published. All authors have read and approved the final manuscript.
